# Editorial: Lessons Learned from Translational Research in Neuromuscular Diseases: Impact on Study Design, Outcome Measures and Managing Expectation

**DOI:** 10.3389/fgene.2022.840074

**Published:** 2022-07-04

**Authors:** Anna G. Mayhew, Leslie Nelson, Michela Guglieri, Tracey Willis

**Affiliations:** ^1^ The John Walton Muscular Dystrophy Research Centre, Translational and Clinical Research Institute, Newcastle University and Newcastle Hospitals NHS Foundation Trust, Newcastle upon Tyne, United Kingdom; ^2^ Department of Physical Therapy, University of Texas Southwestern Medical Center, Dallas, TX, United States; ^3^ Robert Jones and Agnes Hunt Orthopaedic Hospital, NHS Foundation Trust, Oswestry, England

**Keywords:** neuromuscular disorders (NMD), clinical trial, clinical outcome assessments, duchenne muscular dystrophy (DMD), spinal muscular atrophy (SMA), remote assessment

The aim of this research topic is to give the opportunity for clinicians and researchers from around the world to contribute with their experience and knowledge around challenges and solutions in the sphere of translational research in neuromuscular diseases (NMD). This is with reference to study design, outcome measures and what we have learnt from both successful trials and those that did not meet their primary endpoints. Resulting publications around this topic offer exciting and valuable insight which we hope can help guide future clinical trials as well as clinical practice. Lessons learned will benefit those developing research methodology to help improve trial outcomes and therefore clinical care for patients with neuromuscular diseases.

Many factors may contribute to clinical trials not meeting their primary endpoint. One of the identified reasons is the lack of robust data to support the validity of a measure and poor-quality data collection. Significant efforts have been made to ameliorate these issues and one manuscript has provided a framework to guide the learning process for clinical outcome assessments (COAs) for use in both clinics and clinical trials. The process of teaching and learning COAs is necessary to maximize reliability of the measure and validity of the data collected. Evaluators involved in collecting data must do so in a standardised manner and must use clinical judgement and knowledge to accurately perform and score the COA. This manuscript is a consensus-based guideline to assist in the required training that must be employed for study start up for all evaluators regardless of experience (Duong et al.). Traditionally, assessments have been performed in person with direct contact with the research subject. Recent global events have created additional pressures for industry in delivering face to face consultations. A number of industry sponsors quickly introduced amendments to their study protocols to enable remote evaluations. There may be many complications when utilizing a scale initially validated to be performed in person through a remote platform which may potentially impact the scale validity. Here a global network of physiotherapists report on methods for adapting current practices to accommodate remote testing and considerations for remote evaluations (James et al.). Insight from these two manuscripts may provide valuable knowledge for considerations in study design and set up.

Prior experience in clinical trials has provided us another important tool for moving the field forward in these rare diseases. For example, in the last three decades, several compounds have been assessed preclinically and within clinical studies for their ability to restore functional dystrophin levels or to modify pathways involved in Duchenne muscular dystrophy (DMD) pathophysiology. However, there has been a significant attrition rate of early and late phase trials. Here they present data from 16 compounds that failed to complete clinical development, despite positive results in the early phases of development (Markati et al., 2021). The authors examine the reasons for this high failure rate and suggest solutions to ensure the success of future studies. Further to this, additional lessons from trials involving spinal muscular atrophy (SMA) and DMD outline areas in which trial success can be promoted. This paper describes key features including well-defined inclusion criteria, matching criteria, alternative trial designs and the importance of selecting the most appropriate endpoints to ensure a successful trial (Stimpson et al.). And finally, but not insignificantly are the implications of the use of social media in and around clinical trials. Can they be a force for good as well as perhaps their more usually perceived negative impact? In this paper they provide an overview of the guidance that has been published and muse the pros and cons of the use of social media in trials for rare disease (Nelson and Iannaccone). The knowledge based on past experiences may be extremely valuable as we continue to advance clinical trials and clinical care for patients with NMD.

The success of a clinical trial in the rare disease community may be based on many factors. The manuscripts within this issue have touched on some of the aspects that researchers must take into consideration. Furthermore, we must also remember that clinical outcome assessments utilised in clinical trials need to be robust measures (Morel and Cano, 2017) based upon high quality natural history data collected over time (Jacobs et al., 2021). It is not enough that a measure is approved by regulatory authorities, it needs to be meaningful to the patient, sensitive to change and responsive to differences over time. But also taking into consideration that no change for many of these individuals with progressive NMD is important and is perceived as a gain.
